# Trends in Daily Heart Rate Variability Fluctuations Are Associated with Longitudinal Changes in Stress and Somatisation in Police Officers

**DOI:** 10.3390/healthcare10010144

**Published:** 2022-01-12

**Authors:** Herman de Vries, Wim Kamphuis, Cees van der Schans, Robbert Sanderman, Hilbrand Oldenhuis

**Affiliations:** 1 Research Group Digital Transformation, Hanze University of Applied Sciences, Zernikeplein 11, 9747 AS Groningen, The Netherlands; h.k.e.oldenhuis@pl.hanze.nl; 2 Department of Human Behaviour & Training, Netherlands Organization for Applied Scientific Research (TNO), Kampweg 55, 3769 DE Soesterberg, The Netherlands; wim.kamphuis@tno.nl; 3 Department of Health Psychology, University Medical Center Groningen, Antonius Deusinglaan 1, 9713 AV Groningen, The Netherlands; c.p.van.der.schans@pl.hanze.nl (C.v.d.S.); r.sanderman@umcg.nl (R.S.); 4 Department of Rehabilitation Medicine, University Medical Center Groningen, Antonius Deusinglaan 1, 9713 AV Groningen, The Netherlands; 5 Research Group Healthy Ageing Allied Health Care and Nursing, Hanze University of Applied Sciences, Petrus Driessenstraat 3, 9714 CA Groningen, The Netherlands; 6 Department of Psychology, Health and Technology, University of Twente, Drienerlolaan 5, 7522 NB Enschede, The Netherlands

**Keywords:** stress, somatisation, heart rate variability, longitudinal, wearables, ecological momentary assessment

## Abstract

The emergence of wearable sensors that allow for unobtrusive monitoring of physiological and behavioural patterns introduces new opportunities to study the impact of stress in a real-world context. This study explores to what extent within-subject trends in daily Heart Rate Variability (HRV) and daily HRV fluctuations are associated with longitudinal changes in stress, depression, anxiety, and somatisation. Nine Dutch police officers collected daily nocturnal HRV data using an Oura ring during 15–55 weeks. Participants filled in the Four-Dimensional Symptoms Questionnaire every 5 weeks. A sample of 47 five-week observations was collected and analysed using multiple regression. After controlling for trends in total sleep time, moderate-to-vigorous physical activity and alcohol use, an increasing trend in the seven-day rolling standard deviation of the HRV (HRVsd) was associated with increases in stress and somatisation over 5 weeks. Furthermore, an increasing HRV trend buffered against the association between HRVsd trend and somatisation change, undoing this association when it was combined with increasing HRV. Depression and anxiety could not be related to trends in HRV or HRVsd, which was related to observed floor effects. These results show that monitoring trends in daily HRV via wearables holds promise for automated stress monitoring and providing personalised feedback.

## 1. Introduction

Stress can be defined as a relationship between the person and the environment that is appraised by the person as taxing or exceeding one’s resources and endangering one’s well-being [1]. Stress disturbs the body’s biological equilibrium (homeostasis), requiring a neural, neuroendocrine and neuroendocrine-immune adaptation to restore it (allostasis) [2]. Acute stress has a function to trigger a behavioural response to cope with the demand, but chronic stress leads to cumulative wear and tear on bodily systems (allostatic load), which is detrimental to long-term health and well-being [3]. Policing is a good example of a physically and psychologically demanding job that can cause stress [4]. In police officers, chronic stress is associated with neuro-endocrine changes [5] and an increased risk of physical [6], mental illness [7], and absenteeism [8].

The emergence of wearable sensors that allow for unobtrusive monitoring of physiological and behavioural patterns introduces new opportunities to study the impact of stress in a real-world context [9]. In particular, Heart Rate Variability (HRV), which can be measured using wearable sensors, is promising as a biomarker for resilience to stress [10]. If trends in daily HRV observations can be related to mental health outcomes, it enables possibilities for early recognition of the impact of stress and personalised stress counselling based on objective feedback. This study, therefore, explores to what extent daily HRV trends are related to longitudinal changes in several mental health outcomes in police officers. Below, we provide a detailed description of HRV and how its daily fluctuations may be a relevant proxy for homeostatic disturbances, and then we describe this study’s hypotheses.

### 1.1. Heart Rate Variability (HRV)

HRV is a measure for the variation in inter-beat-intervals that reflects autonomic nervous system functioning and is negatively correlated to allostatic load [11]. HRV declines during stress [12] and can remain suppressed during subsequent rest and sleep [13]. Originally, HRV was measured using electrocardiography (ECG), but in recent years, wearable sensor technologies increasingly started using photoplethysmography (PPG). Unlike ECG, which is based on the electrocardiographic signal that is related to the contraction of the heart muscle, PPG quantifies HRV by assessing the blood flow in peripheric arteries to assess heart rate. Due to this subtle difference, PPG-based HRV is sometimes referred to as “Pulse Rate Variability” [14], but it has been shown to estimate HRV and mental states, such as stress and anxiety [15,16].

HRV can be seen as a resource that enables cognitive and emotional regulation [17] and is physiologically depleted when dealing with demands [18]. Since HRV is associated with stress-buffering effects [19,20], its depletion may indicate a decline in resilience to cope with new demands and thus lead to unfavourable outcomes if a trend develops. This aligns with the conservation of resources theory, which suggests that an initial loss of resources can create a negative feedback loop that results in a loss spiral [21].

Longitudinal studies showed that a decline in HRV can be related to increased stress [22,23,24,25] or emotional exhaustion [26,27,28]. HRV-related emotional exhaustion is often interpreted in the context of burnout but can overlap with depression [29], suggesting that changes in HRV may also be related to other mental health outcomes. Although longitudinal evidence for relationships between HRV and other mental health and well-being outcomes is limited, population studies have shown that HRV is indeed not just negatively associated with stress [30] but also with anxiety [31], depression [32], and somatic symptoms [33]. The overlap in the associations between these diverse mental health and well-being outcomes with HRV is the result of their similar negative impact on autonomic nervous system functioning (decreased vagal tone), of which HRV is a reflection [34]. Since changes in HRV are therefore not a direct proxy for any specific mental state, using a broad approach when investigating the relationship between structural changes in HRV and diverse mental health and well-being outcomes is warranted.

Since most of the existing evidence is based on cross-sectional population studies, recent reviews on HRV literature call for future studies with a longitudinal and within-subject focus [30,35]. Traditional longitudinal studies assess HRV by taking one- or multi-day samples across a period of weeks, months or years, but wearables can unobtrusively collect HRV data on a daily basis. An academic study of this more granular HRV data may help in obtaining a better understanding of relationships between HRV and other variables in a naturalistic setting. These more granular data allow us to look at trends in daily HRV but also open up the possibility to assess the degree to which the daily HRV fluctuates over time.

### 1.2. Daily HRV Fluctuations

Since HRV reflects autonomic nervous system functioning, daily HRV fluctuations can be seen as a proxy for the homeostatic disturbances that form an allostatic burden. The autonomic nervous system continually strives to restore homeostasis. As such, it is possible that homeostatic disturbances exist while the average level of physiological functioning itself (e.g., the mean HRV) remains relatively constant. In this scenario, the allostatic process that continuously restores homeostasis is successful, but the pressure on the system as a whole may still be indicative of underlying problems.

Research on associations between daily HRV fluctuations and stress is still nascent in occupational settings, but interesting parallels to sports science can be drawn. For instance, increases in daily HRV fluctuations have consistently been associated with increased fatigue in athletes [36,37,38] but were also attributed to increased stress in a study describing a notable case of a female soccer player [39]. Another study found that soccer players that had a decreased HRV and increased daily HRV fluctuations after a high-load week had a decreased stress tolerance [40], suggesting that these homeostatic disturbances may also impact the individuals’ resilience to cope with upcoming demands. Therefore, changes in daily HRV fluctuations may be an interesting precursor to identify the development of more structural changes in stress.

As a result, trends and fluctuations in daily HRV may not only be directly related to changes in mental health outcomes, but trends in the underlying daily HRV itself may also moderate that association. An example of this was presented in a case study under elite triathletes. In the study, a decrease in daily HRV fluctuations, which is usually seen as a sign of positive adaptation, preceded poor performance and subsequent illness when the downtrend in daily fluctuations was combined with a downtrend in the daily HRV itself [41]. The decrease in daily HRV fluctuations was not a sign of positive adaptation in this case but may actually have been indicative of a lack of autonomous nervous system reactivity to the challenges at hand since the underlying daily HRV was also trending down. Conversely, it is also possible that an uptrend in the daily HRV may buffer against the unfavourable effect of uptrends in daily HRV fluctuations on relevant outcomes. In that scenario, an uptrend in the daily HRV fluctuations would indicate that the individual’s homeostasis is increasingly being challenged, but the uptrend in the daily HRV itself shows that the underlying physiological system itself is actually responding resiliently.

### 1.3. Aim of the Study

Existing literature showed that a longitudinal decrease in HRV is positively associated with increased stress and that having a lower HRV is related to increased depression, anxiety and somatisation at a population level. There are also indications that increases in daily HRV fluctuations are related to unfavourable outcomes and that an increasing daily HRV trend could have a buffering effect. Therefore, this study aims to explore to what extent within-subject trends in daily HRV and daily HRV fluctuations are related to changes in stress, depression, anxiety, and somatisation in police officers in a large Dutch city. By applying a longitudinal design that utilises continuous daily measurements in a real-world context, this study provides a unique contribution to the existing body of knowledge. We hypothesise that increasing trends in daily HRV fluctuations and decreasing trends in daily HRV are associated with increased (1) stress, (2) depression, (3) anxiety, and (4) somatisation, as well as that increasing trends in daily HRV buffer against the positive association between trends in daily HRV fluctuations and these outcomes.

## 2. Materials and Methods

The study protocol was approved by the ethical committee of the Hanze University of Applied Sciences Groningen (heac.2020.012).

### 2.1. Participants

Police officers that worked in a large Dutch city and possessed a smartphone running on Android or iOS were invited to participate. The officers received an information letter via e-mail that informed them about the study. Participants were asked to collect data for 15 weeks, with an option to extend this to 20 weeks to reach a reward threshold, but participated voluntarily and were free to stop at any time. Participants that collected complete daily data on at least 100 days (>71–95% adherence based on a period of 15–20 weeks) and completed all longitudinal questionnaires were allowed to keep the wearable and received a feedback report after the study. Recruitment started in June 2020 and was completed in July 2020 after reaching the capacity of 10 participants, which was related to the availability of materials. Participants gave their informed consent prior to participation and had a conversation with the first author before and after their data collection period. Due to policies related to the COVID-19 pandemic, which was ongoing during data collection, these conversations were held via a teleconferencing tool. One participant was excluded from analysis due to diagnosed atrial fibrillation. The remaining 9 participants were predominantly male (77.8%) and had mean age of 35.8 years (25.8–51.1).

### 2.2. Data Collection

Data collection started after the participants received their wearable and was planned to run for 15 to 20 weeks. Some participants voluntarily extended this period. During data collection, participants collected daily wearable data and a daily Ecological Momentary Assessment (EMA) question and filled in a longitudinal questionnaire every 5 weeks. Participants, therefore, collected multiple nested five-week observations. All participants reached the reward threshold. One participant collected data for 15 weeks, five for 25 weeks, whereas three participants proceeded to collect data for 25, 40, and 55 weeks. As a result, change scores and trends in the corresponding daily measurements were calculated for a sample of 47 five-week observations that were analysed.

#### 2.2.1. Stress, Anxiety, Depression and Somatisation

The Dutch version of the Four-Dimensional Symptom Questionnaire (4DSQ) was used to measure stress, anxiety, depression, and somatisation every 5 weeks [42]. The 4DSQ consists of 50 items, of which 16 concerning stress, 12 on anxiety, 5 on depression, and 16 on somatisation. All items inquire about the occurrence of symptoms over the previous week and are scored on a 5-point Likert scale (‘no’, ‘sometimes’, ‘regularly’, ‘often’, or ‘very often or constantly’). Responses are scored as 0 (‘no’), 1 (‘sometimes’), or 2 (‘regularly’, ‘often’, or ‘very often’) and summarised to create the overall scores on each scale. Each scale has cut-off points for moderately or severely elevated levels for clinical use, but these were not used for data analysis in this study. Five-week change scores were calculated by subtracting the scores of the 4DSQ scales from the scores on the subsequent measurement, resulting in 4 variables in which a higher score indicates an increase in the measured concept: stress increase, anxiety increase, depression increase, and somatisation increase.

#### 2.2.2. Daily HRV & Daily HRV Fluctuations

Daily HRV was measured with an Oura ring during sleep. The Oura ring is a consumer-available wearable with the size of a wedding ring, has a battery life of 4–7 days and measures sleep, physical activity, temperature, heart rate, and HRV. In this study, a second-generation Oura ring was used, which uses infrared light to measure HRV via PPG. The Oura ring uses a built-in artefact identification algorithm that is described in more detail elsewhere [43]. In short, the algorithm in the ring labels each inter-beat-interval (IBI) as normal or abnormal by calculating its deviation from the median of the nearest surrounding IBIs. The night is then subdivided into 5-min segments for which the HRV is calculated. Finally, the average HRV of all 5-min segments that have sufficient valid measurements is then calculated to obtain the HRV for the full night. A validation study under 49 healthy individuals aged 15–72 years showed that the Oura ring’s HRV measure explained 98.0% of the variance (r^2^ = 0.980) in the gold standard ECG-based HRV measurement [43]. Another study under 5 healthy young adults that generated 23 trials found that the Oura ring had the second-lowest mean absolute percent error of the 7 investigated consumer-available wearables and reported a 0.91 correlation coefficient with ECG measurements [44]. Participants in this study selected a ring type and colour of their preference with an optimal fit for both user comfort and measurement accuracy. To preserve privacy, anonymised Oura accounts were created. The HRV metric reported by the Oura ring is the Root Mean Square of the Successive Differences (RMSSD), which is a time-domain metric for vagally mediated HRV and is expressed in milliseconds [45]. To improve the distribution for statistical modelling, the RMSSD was logarithmically transformed (lnRMSSD), which is a common procedure [46].

Daily HRV fluctuations were operationalised by calculating the 7-day rolling standard deviation (HRVsd) when HRV observations on at least three out of the seven prior days were available [47]. Other studies on daily HRV fluctuations have applied a seven-day moving window to account for weekly influences and calculate a coefficient of variation [37,41]. Using the coefficient of variation is helpful if between-subject comparison of HRV fluctuations is targeted, as HRV can differ vastly between individuals [48]. That approach does not apply to this study, which analyses within-subject trends in daily HRV and fluctuations therein. Since this study explores moderating effects between those two trends, using a coefficient of variation means that a small portion of the variation of the daily HRV trend is included in the daily HRV fluctuations metric, increasing the likelihood of a type II error occurring and making it less ideal than using a seven day rolling standard deviation within the aims of this study.

To determine trends in daily HRV, HRVsd, and control variables, a linear regression model was used to examine the rate of change as a function of time [41]. To do so, measurements between longitudinal questionnaires were first filtered into subsets with the daily observations between two questionnaires. Linear regression models were then created by regressing each of the variables on time (the dates). A positive beta-coefficient, therefore, represents an uptrend over the respective period, whereas a negative beta-coefficient represents a downtrend.

#### 2.2.3. Control Variables

To account for confounders, control variables for Total Sleep Time (TST), Moderate-to-Vigorous Physical Activity (MVPA), and alcohol use were included. TST, which is the total duration of the main sleep episode that the user is asleep, was measured using the Oura ring [49]. The Oura ring also measured MVPA, which is the number of minutes of physical activity at an intensity of at least 3 times the metabolic equivalent (MET) of the resting state. Alcohol use was measured with a daily EMA question that was available from 19:00 to 15:00 the next day to accommodate for night shifts. The item inquired about the number of consumed alcoholic beverages that day and was based on the AUDIT-C questionnaire [50]. Data for the EMA question were collected using an in-house developed smartphone application and stored on-premise. As with HRV and HRVsd, trend scores were determined via linear regression models, where a positive beta-coefficient represents an uptrend, and a negative beta-coefficient represents a downtrend on the measure within the respective five-week period.

### 2.3. Data Analysis

Data management and analyses were performed in RStudio [51] and R [52]. Values for the changes on the longitudinal questionnaires and trends in the daily observations were standardised at the grand mean (subtracting the mean value of all observations from each value and then dividing it by the standard deviation of all observations). This procedure was applied to prevent scaling issues during statistical testing and optimise the comparability of the beta-coefficients of the final models [53]. Each five-week data collection period represented one observation for which the change scores on the longitudinal questionnaire are compared to the trends in the daily measurements. For instance, if all 10 participants completed 4 data collection periods, that would result in a total sample of 40 observations available for analysis.

A three-step hierarchical modelling approach was used. The outcomes were first modelled based on the control variables for trends in TST, MVPA, and alcohol use, after which the main variables for trends in HRV and HRVsd were added in step two. In the third and final step, the interaction effect between trends in HRV and HRVsd was added to create the full model. Initially, Linear Mixed Modelling with fixed effects and random slopes was performed to account for repeated measurements within participants. However, analyses experienced singular bound problems due to a lack of between-subject variance, which is a sign that the fitted models may be too complex and more parsimonious models should be considered [54,55]. We, therefore, chose to apply more parsimonious multiple regression analyses instead, which yielded the same results and conclusions that were drawn based on the initial multi-level modelling approach.

## 3. Results

The data of this sample of 47 five-week observations included a total of 57 longitudinal questionnaires. Daily data were available on 1734 unique person-days, of which 1648 (94.3%) included HRV data and 1458 (89.0%) included EMA data. Based on interviews and manual inspection of missing data, missing HRV data were attributed to drained ring batteries, accidentally not wearing the ring, or could not be explained. Missing EMA questionnaire data were primarily attributed to forgetting to fill it in before going to bed. Two participants (22.2%) had moderately elevated stress on the 4DSQ at baseline, whereas the remaining seven (77.8%) did not have elevated stress at baseline. No participants (0%) had elevated depression, anxiety, or somatisation levels at baseline. The intercorrelations between the changes in the longitudinal questionnaires and trends in the wearable and control variables are described in Table 1.

**Figure 1:**
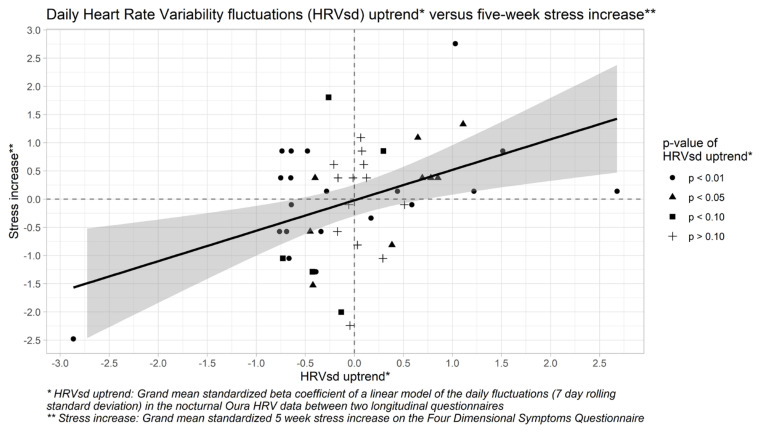
Scatter plot for the five-week uptrends in the 7-day rolling standard deviation of the Heart Rate Variability (HRVsd) versus five-week stress increases on the Four-Dimensional Symptom Questionnaire (4DSQ) in all 47 observations of the 9 participants in this study. The grey area represents the 95% confidence interval for the values that are estimated by the linear model (the thick black line).

A three-step hierarchical multiple regression model for five-week stress changes was formed (Table 2). After controlling for trends in TST, MVPA, and alcohol use, uptrends in daily HRVsd were associated (*p* = 0.004) with increased stress, whereas daily HRVsd downtrends were associated with decreased stress (Figure 1). Trends in the daily HRV itself were unrelated to stress and did not buffer against the positive association between trends in daily HRVsd and stress increases. Hypothesis 1 is therefore partially confirmed. The full model explains 18.5% of the variance in five-week stress changes and provides a marginally significant improvement (*p* = 0.08) over the control model but is not significantly different from the main effects model (*p* = 0.96).

**Figure 2:**
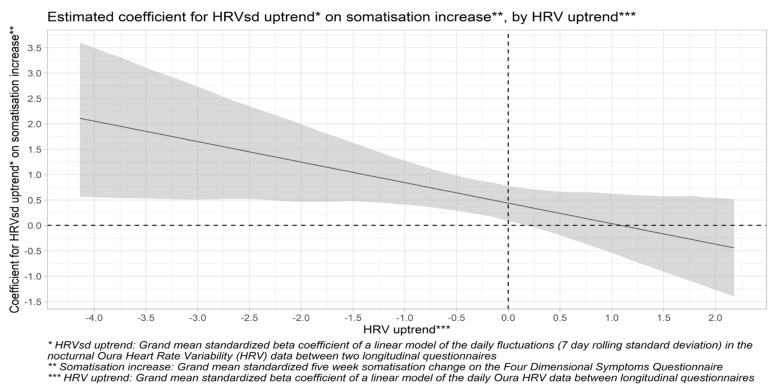
Estimated coefficient for the association between the five-week uptrend in the 7-day rolling standard deviation of the Heart Rate Variability (HRVsd) and five-week somatisation increase by the five-week HRV uptrend. The grey area represents the 95% confidence interval for the values that are estimated by the linear model (the thick black line).

Another three-step hierarchical multiple regression model was formed for a five-week somatisation change (Table 3). After controlling for trends in TST, MVPA, and alcohol use, uptrends in daily HRVsd were positively associated (*p* = 0.01) with somatisation increase, whereas downtrends in daily HRVsd were associated with a decrease in somatisation. Trends in daily HRV itself were not associated with changes in somatisation, but uptrends in daily HRV moderated (*p* = 0.04) the association between daily HRVsd uptrends and somatisation increase. The interaction plot in Figure 2 shows that uptrends in daily HRVsd are associated with somatisation increase when the daily HRV trends down, but not when it trends up. Therefore, uptrends in daily HRV buffer against the positive association between uptrends in daily HRV fluctuations and somatisation increase, as expected. Hypothesis 4 is therefore partially confirmed. The full model explains 21.3% of the variance in the five-week somatisation change and provides a marginally significant improvement (*p* = 0.07) over the control model but not over the main effects model (*p* = 0.76).

For depression (Hypothesis 2) and anxiety (Hypothesis 3), no models could be formed. This was related to floor effects on both scales. On the 56 questionnaires, 52 (92.9%) were scored zero on depression and 49 (87.5%) on anxiety. None reached cut-off points for elevated levels, illustrating a complete absence of clinically relevant symptoms.

## 4. Discussion

This study hypothesised that increasing trends in daily HRV fluctuations and decreasing trends in daily HRV are associated with five-week increases in (1) stress, (2) depression, (3) anxiety, and (4) somatisation, and that increasing trends in daily HRV buffer against the positive association between the uptrends in daily HRV fluctuations and increases in these outcomes. The results of this study showed that uptrends in daily HRV fluctuations were indeed associated with increased stress and somatisation, and uptrends in daily HRV buffered against the positive association between uptrends in daily HRV fluctuations and somatisation increase (Hypotheses 1 and 4). Uptrends in daily HRV were not directly associated with changes in any of the outcomes, and changes in depression and anxiety could not be linked to trends in daily HRV nor daily HRV fluctuations (Hypotheses 2 and 3) due to floor effects. Hypotheses 1 and 4 are therefore partially confirmed, whereas Hypotheses 2 and 3 are unconfirmed.

### 4.1. Associations between Daily HRV Fluctuations, Stress and Somatisation

When the day-to-day variation in the daily HRV trended up, participants were more likely to report increased stress on the next five-weekly questionnaire. Since HRV reflects the functioning of the autonomic nervous system [56], which continuously strives to restore homeostasis when faced with stress [2], the relationship between increased stress and homeostatic disturbances is intuitive. Although the existing body of knowledge on this topic is limited, this result aligns with that of two prior studies that related an uptrend in daily HRV fluctuations to stress increase in soccer players [39,40]. To our knowledge, this study is the first to explore associations between trends in daily HRV and fluctuations therein to longitudinal mental health outcomes in an occupational setting. The reported results, therefore, contribute valuable new insights that measuring HRV on a daily basis using a consumer-available wearable may be a feasible and effective approach for the unobtrusive and early recognition of changes in stress in occupational settings.

This study also linked uptrends in daily HRV fluctuations to increased somatisation scores, whereas daily HRV uptrends were buffered against this. The interaction plot in Figure 2 showed that the association between uptrends in daily HRV fluctuations and increases in somatisation is only significant when combined with a downtrend in daily HRV but not when daily HRV is increasing. To our knowledge, this specific association has not been addressed in prior literature. There is some overlap with a prior study that reported an association of increased daily HRV fluctuations and decreased daily HRV with muscle soreness in swimmers during overload training [57]. However, in our study, somatisation increase was not related to uptrends in MVPA (Table 3) but was significantly correlated (r = 0.56; Table 1) to stress increase, underlining a possible difference in the underlying mechanisms between training- and stress-induced somatic symptoms.

### 4.2. Floor Effects in Depression and Anxiety

No associations of trends in daily HRV and daily HRV fluctuations with five-week changes in depression and anxiety were found. Since 92.9% of all depression scores and 87.5% of all anxiety scores were zero and the cut-off points for elevated levels on these scales were not reached on any observation, there was a complete absence of clinically relevant symptoms on these dimensions. Since the presence of floor effects means that there may be insufficient variance within the respective sample to find a statistically significant effect even if there could be one in the full population (type II error) [58], this absence of proof should not be interpreted as a proof of the absence of this association. Based on population studies that related HRV to depression [32] and anxiety [31], this hypothesis warrants further investigation in future studies under populations that experience more clinically relevant symptoms or that use more sensitive measurement instruments.

### 4.3. Strengths and Limitations

This study used a consumer-available wearable that is known to produce valid daily HRV data [43,44] to measure HRV on 94.3% of all 1734 person-days on which data were reported. The trends in daily HRV that are analysed in this paper are therefore based on more granular data than longitudinal studies that apply a pretest-posttest design and are likely a good reflection of the true daily HRV trends over the full five-week periods. Another strength of this study is that data were collected within a naturalistic setting, optimising the generalisability of the reported findings to real-world applications. By applying a novel design to assess the relationship between daily HRV observations that are measured with a consumer-available wearable and longitudinal mental health outcomes, this study takes important steps in a nascent but promising research field.

A sample of 47 five-week observations was analysed, based on data collected by nine Dutch police officers. Not all participants contributed equally in the number of collected five-week observations. Although there are no indications that using nested observations within this sample or the unequal contribution of observations were problematic, replication of these findings in a larger sample of participants collecting an equal number of observations would be beneficial for the external validity. Similarly, future studies that analyse a larger number of observations may consider applying cross-validation methods. For example, studies could use the observations of a subset of participants to predict the outcomes in the remainder of the participants.

Finally, data collection occurred during the COVID-19 pandemic. A study under 2567 European police officers reported decreased strain during the pandemic, where the risk of infection and deficient communication were the main stressors [59]. Another study found that COVID-19 lockdowns lead to increased HRV in 20% and decreased HRV in 80% of the French general population [60]. Thus, it is possible that the unique context of this period influenced the observed daily HRV values and mental health outcomes. However, since this study does not assess these actual outcomes but observes to what extent trends between them are interrelated, this context is unlikely to have directly influenced this study’s findings.

### 4.4. Implications

The results showed that uptrends in daily HRV fluctuations were related to five-week increases in stress and somatisation and that uptrends in daily HRV buffer against the association between uptrends in daily HRV fluctuations and somatisation increase. If these findings are replicated in future studies, they show that tracking daily HRV using a consumer-available wearable holds promise for early recognition of the impact of stress and for personalised feedback based on objective data in stress management interventions. Companies that are developing these wearable devices or related systems can then consider including metrics for daily HRV fluctuations (e.g., the 7-day rolling standard deviation or coefficient of variation) and estimate periodic trends in the daily HRV and daily HRV fluctuations. The presence of a statistically significant trend in the daily HRV fluctuations could then be used to trigger personalised in-app feedback, notifying the user that a trend was witnessed that may be stress-related. Such a trigger could, for instance, nudge the user to reflect on the current situation, consider coaching or offer other interventions aiming to limit the negative impact of stress.

## Figures and Tables

**Table 1:** Intercorrelations between the wearable (1–2), longitudinal (3–6), and control (7–9) variables.

Variable	Correlation							
	1	2	3	4	5	6	7	8
1. HRV uptrend	−							
2. HRVsd uptrend	−0.04	−						
3. Stress increase	−0.09	0.43 **	−					
4. Anxiety increase	−0.00	−0.04	0.24	−				
5. Depression increase	0.06	−0.03	0.31 *	0.15	−			
6. Somatisation increase	−0.03	0.42 **	0.56 ***	−0.03	0.09	−		
7. TST uptrend	0.01	−0.01	0.11	−0.22	0.10	0.09	−	
8. MVPA uptrend	−0.13	0.09	−0.21	−0.05	0.12	−0.17	−0.28	−
9. Alcohol use uptrend	−0.12	−0.21	−0.06	0.28	0.03	−0.19	−0.47 ***	0.14

Note. N = 47; *** *p* < 0.001, ** *p* < 0.01, * *p* < 0.05, *p* < 0.1; HRV: Heart Rate Variability; HRVsd: Heart Rate Variability, 7-day rolling standard deviation; TST: Total Sleep Time; MVPA: Moderate-to-Vigorous Physical Activity.

**Table 2:** Hierarchical multiple regression model for five-week stress increase.

	Stress Increase				
	Step 1	Step 2		Step 3	
Independent Variable	β	β		β	
Intercept	−0.019	−0.024		−0.029	
TST uptrend	0.048	0.075		0.056	
MVPA uptrend	−0.209	−0.275		−0.276	
Alcohol use uptrend	−0.005	0.100		0.092	
HRV uptrend		−0.098		−0.089	
HRVsd uptrend		0.590	**	0.542	**
HRV uptrend * HRVsd uptrend				−0.224	
R^2^	0.047	0.267		0.291	
Adjusted R^2^	−0.019	0.177		0.185	
F	0.711	2.984	*	2.737	*
ΔR^2^		0.220		0.024	
ΔF		2.273		−0.247	

Note. N = 47; ** *p* < 0.01, * *p* < 0.05, *p* < 0.1; HRV: Heart Rate Variability; HRVsd: Heart Rate Variability, 7-day rolling standard deviation; TST: Total Sleep Time; MVPA: Moderate-to-Vigorous Physical Activity.

**Table 3:** Hierarchical multiple regression model for five-week somatisation increase.

	Somatisation Increase				
	Step 1	Step 2		Step 3	
Independent Variable	β	β		β	
Intercept	0.003	−0.002		−0.012	
TST uptrend	−0.051	−0.024		−0.058	
MVPA uptrend	−0.169	−0.224		−0.226	
Alcohol use uptrend	−0.191	−0.091		−0.107	
HRV uptrend		−0.054		−0.038	
HRVsd uptrend		0.530	**	0.443	*
HRV uptrend * HRVsd uptrend				−0.407	*
R^2^	0.061	0.234		0.315	
Adjusted R^2^	−0.004	0.141		0.213	
F	0.931	2.508	*	3.069	*
ΔR^2^		0.173		0.081	
ΔF		1.577		0.561	

Note. N = 47; ** *p* < 0.01, * *p* < 0.05, *p* < 0.1; HRV: Heart Rate Variability; HRVsd: Heart Rate Variability, 7-day rolling standard deviation; TST: Total Sleep Time; MVPA: Moderate-to-Vigorous Physical Activity.

## Data Availability

Data of this article are not publicly available due to the personal nature of the data in combination with the sensitive professions of the participants of this study.
